# Higher galectin-3 levels are independently associated with lower anxiety in patients with risk factors for heart failure

**DOI:** 10.1186/s13030-020-00195-7

**Published:** 2020-10-02

**Authors:** Monika Sadlonova, Thomas Meyer, Lutz Binder, Rolf Wachter, Frank Edelmann, Christoph Herrmann-Lingen

**Affiliations:** 1 Department of Psychosomatic Medicine and Psychotherapy, University of Göttingen Medical Center, Göttingen, Germany; 2 Department of Thoracic and Cardiovascular Surgery, University of Göttingen Medical Center, Göttingen, Germany; 3 German Center for Cardiovascular Research (DZHK), partner site Göttingen, Göttingen, Germany; 4 Institute for Clinical Chemistry, University of Göttingen Medical Center, Göttingen, Germany; 5 Department of Cardiology and Pneumology, University of Göttingen Medical Center, Göttingen, Germany; 6 Department of Cardiology, University of Leipzig Medical Center, Leipzig, Germany; 7 Department of Internal Medicine and Cardiology, Charité University Medicine, Campus Virchow Klinikum, Berlin, Germany; 8 German Center for Cardiovascular Research (DZHK), partner site Berlin, Berlin, Germany; 9 Berlin Institute of Health (BIH), Berlin, Germany

**Keywords:** Galectin-3, Anxiety, Cardiovascular risk factors

## Abstract

**Background:**

Galectin-3 promotes the proliferation of neural progenitor cells and is engaged in cell-cell adhesion, cell-matrix interactions, and macrophage activation. In addition, in patients with heart failure this carbohydrate-binding protein is a known prognostic marker for cardiovascular mortality. However, its association with psychological variables has not been investigated so far.

**Methods:**

Using data from the multicenter, observational Diast-CHF (Diagnostic Trial on Prevalence and Clinical Course of Diastolic Dysfunction and Heart Failure) trial, we studied in participants with cardiovascular risk factors (*n* = 1260, age 66.7 ± 8.0 years, males 51%, left ventricular ejection fraction 60.0 ± 8.1%) the relationship between serum concentrations of galectin-3 and anxiety. Galectin-3 levels were measured by means of a sandwich enzyme-linked immunosorbent assay, and anxiety was assessed using the Hospital Anxiety and Depression Scale (HADS).

**Results:**

In univariate analysis, there was a weak but significant inverse correlation between galectin-3 and HADS anxiety (rho = − 0.076; *p* = 0.008). Linear regression models adjusted for sex, age, body-mass index, estimated glomerular filtration rate, left ventricular ejection fraction, 6-min walking distance, the 36-item Short-Form Health Survey (SF-36) subscale physical functioning, and known biomarkers for heart failure confirmed that serum galectin-3 significantly and independently predicted self-rated anxiety (B = -2.413; 95%CI = -2.413–-4.422; *p* = 0.019).

**Conclusion:**

In patients with cardiovascular risk factors, serum concentrations of galectin-3 showed an inverse association with anxiety, which was independent of both the severity of physical impairment and established risk factors for the progression of heart failure.

## Background

In patients with cardiac diseases, the impact of anxiety on prognosis has been controversially discussed in the literature. While only two observational studies reported a protective effect of anxiety on mortality in patients with coronary heart disease, numerous studies demonstrated that anxiety disorders are associated with increased mortality and worse prognosis [1–8]. In patients with heart disease, several parameters of neuroendocrine activation and inflammation have been found increased and some of these markers, such as natriuretic peptides or C-terminal pro-arginine vasopressin (CT-proAVP), have been proposed to elicit anxiolytic effects [9–16]. Galectin-3 has been introduced as another prognostic biomarker for increased mortality risk in heart failure [17–24]. This 35 kDa protein belongs to the family of β-galactoside-binding lectins with a carboxyl-terminal lectin domain connected to an amino-terminal non-lectin segment including several repeats of a proline/tyrosine/glycine-rich motif [25, 26]. It is expressed in inflammatory processes by mast cells and macrophages, where it regulates cell-cell adhesion, cell-matrix interactions, proliferation, angiogenesis, and induction of pre-mRNA splicing [27, 28]. The secretion of galectin-3 activates fibroblasts and myofibroblasts to secrete procollagen into the extracellular matrix as a component in the cardiac remodeling process [29–31]. However, possible associations of galectin-3 with emotional well-being in general, and anxiety in particular, are unclear.

Sartim et al. reported that galectin-3 knockout mice had increased compulsive-like behavior and, in addition, showed higher immobility times in the forced swim test and tail suspension test, suggesting an impaired stress-coping behavior [32]. Notably, Stajic and colleagues showed that galectin-3-deficient mice displayed anxiogenic effects in mature untreated animals compared to wild-type animals, and this was accompanied by reduced interleukin-6 (IL-6) and tumor necrosis factor-α (TNF-α) gene expression and protein content in the hippocampus [33]. However, in humans, specific associations of galectin-3 with anxiety have not been studied so far. To address the question whether serum levels of galectin-3 are associated with anxiety, we used data from the population-based, multicenter Diagnostic Trial on Prevalence and Clinical Course of Diastolic Dysfunction and Heart Failure (Diast-CHF) study and looked for associations of the serum galectin-3 measurements with self-assessed anxiety in subjects presenting with a cardiovascular risk profile.

## Methods

### Study design

The baseline assessment of the prospective, multicenter Diast-CHF trial was conducted in the years 2004 to 2006 as part of the nationwide German Competence Network Heart Failure, which aimed at assessing the prevalence of diastolic left ventricular dysfunction in medical outpatients treated by primary care physicians [34]. The participants in this observational study were 1727 outpatients aged 50–85 years with cardiovascular risk factors and/or manifest heart failure. An additional control group of 208 healthy volunteers was excluded from the present analysis, as were 49 participants with known atrial fibrillation and 136 subjects lacking echocardiographic parameters for diastolic dysfunction. Due to the lack of galectin-3 measurements and incomplete data on psychometric assessment, another 282 participants were excluded. In the end, the study population analyzed in this work comprised 1260 participants with complete data. Medical outpatients were eligible as study participants if they had at least one cardiovascular risk factor for the development of diastolic heart failure, such as diabetes mellitus, coronary heart disease, hypertension, sleep apnea, history of myocardial infarction, or manifest heart failure. Exclusion criteria were insufficient understanding of the German language, unwillingness to give informed consent and/or unavailability for logistic reasons. All participants received routine physical examination and a detailed echocardiogram for the assessment of systolic and diastolic function, including the American Society of Echocardiography (ASE) grade and ejection fraction (LVEF). Before being included in the study, all patients gave their written informed consent. The study protocol was approved by all local institutional ethics committees and complies with the Declaration of Helsinki.

### Assessment of anxiety and quality of life

Study participants were requested to complete the German version of the Hospital Anxiety and Depression Scale (HADS) [35]. This short self-rating questionnaire, initially developed for the assessment of both anxiety and depression in physically ill patients, comprises 14 four-point Likert-scaled items on two subscales, with 7 items each for anxiety and depression [36]. The severity of symptoms is rated on scales ranging from 0 to 3 for each item, and the values achieved for each item are then added, so that total values from 0 to 21 can be reached per subscale. The HADS including its German version has been validated as a measure of anxiety, and a good reliability with Cronbach’s alpha of 0.80 has been reported [35]. Individual dimensions of health-related quality of life were measured using the 36-item Short-Form Health Survey (SF-36), which was first evaluated in the Medical Outcome Study and has since then emerged as one of the most widely validated generic quality of life instruments [37].

### Laboratory measurements

From each study participant, venous blood samples were drawn and collected in tubes from the antecubital vein after 15 min of rest in a prone position. Heparinized and EDTA samples were immediately obtained by centrifugation, and supernatants were subsequently stored at − 80 °C, until they were analyzed in a specialized laboratory. The measurements of galectin-3 were performed using a sandwich-ELISA (enzyme-linked immunosorbent assay) from BG Medicine, Waltham, MA/USA. This immunoassay used a rat and a mouse monoclonal antibody raised against a mouse galectin-3 protein [38, 39]. The measuring and linearity range of galectin-3 was given as 1.4–94.8 ng/mL for clinical specimens. The total coefficient of variation was 12% at a concentration of 6.1 ng/mL, and 7.7% at 20.7 ng/mL. The 90^th^, 95^th^ and 97.5^th^ percentiles in an apparently healthy population were 17.6 ng/mL, 20.3 ng/mL and 22.1 ng/mL, respectively [39]. In healthy subjects aged 55 years or more, the 95% range interval was from 3.8 to 21 ng/mL [38]. In patients with decompensated heart failure, the concentration of galectin-3 is between 4 to 75 ng/mL with a cutoff value of 22.1 ng/mL.

Since we previously observed an inverse association of natriuretic peptides and CT-proAVP with anxiety in the same cohort, we additionally controlled for natriuretic peptides and CT-proAVP in the present analysis [14, 16]. N-terminal pro-brain natriuretic peptide (NT-proBNP) was measured with a non-competitive electro-chemiluminescence immunoassay (Elecsys, Roche Diagnostics, Mannheim, Germany) using a monoclonal biotinylated NT-proBNP antibody directed against amino acids 1–21 and a secondary monoclonal antibody conjugated to a ruthenium complex binding to amino acids 39–50 [40, 41]. Mid-regional MR-proANP was determined in EDTA plasma using an immunoluminometric assay (SERISTRA from B.R.A.H.M.S) with two polyclonal affinity-purified sheep antibodies directed against different peptide fragments of proANP [42]. The biomarker CT-proAVP was measured with a commercially available chemiluminescence sandwich assay (B.R.A.H.M.S., Hennigsdorf, Germany) using antibodies against the two peptides PATV17 and PLAY17 [43, 44].

### Statistical analysis

Demographic and clinical data are presented as means and standard deviations or frequencies and percentages, while plasma levels of galectin-3 and other biomarkers are given as medians and 25^th^ and 75^th^ percentiles. The biomarkers were logarithmically transformed prior to further analysis in order to approach normal distribution, which was tested using the Shapiro-Wilk test. In general, laboratory variables were not distributed normally even after log transformation. In view of this lack of normal distribution, we used Spearman’s correlation for examining the bivariate relationship between galectin-3 and HADS anxiety. A linear regression model was computed with HADS anxiety as dependent variable adjusted for sex, age, body-mass index (BMI), estimated glomerular filtration rate (eGFR), left ventricular ejection fraction (LVEF), American Society of Echocardiography (ASE) grade of diastolic dysfunction, 6-min walking distance, and SF-36 physical functioning, while plasma galectin-3 was additionally entered as an independent variable (model 1). These variables were selected based on our previous publication in order to maintain the results comparable [16]. In a second model, plasma NT-proBNP, MR-proANP and CT-proAVP were entered as additional confounders. All analyses were performed using SPSS software, version 25 (SPSS Inc., Chicago, IL, USA). A *p* value of < 0.05 was considered statistically significant.

## Results

Approximately half of the study population were men (51.0%), and the mean age of the participants was 66.7 ± 8.0 years (Table 1). Due to the inclusion criteria, the majority of all patients (82.7%) were suffering from echocardiographically documented diastolic dysfunction, as judged by an American Society of Echocardiography grade of ≥ 1. Less than 7% of all participants were suffering from heart failure symptoms with New York Heart Association (NYHA) class II or higher. The most frequent cardiovascular risk factor was arterial hypertension, which was observed in 1118 participants (88.7%). In the total study cohort, the mean HADS anxiety score was 5.1 ± 3.7. The median galectin-3 concentration was 11.7 ng/mL (25^th^ and 75^th^ percentile: 9.9 and 14.1ng/mL), and the median plasma level of NT-proBNP was 101.4 pg/mL (25^th^ and 75^th^ percentile: 52.5 pg/mL and 193.1 pg/mL). For MR-proANP, the median concentration was 91.9 pmol/L (25^th^ and 75^th^ percentile, 64.4 pmol/L and 135.1 pmol/L), whereas for CT-proAVP this was 4.1 pmol/L (25^th^ and 75th percentile, 2.6 and 7.6 pmol/L). The mean HADS score gradually decreased from the first and lowest quartile of serum galectin-3 (5.75) over the second quartile (5.09) to the third quartile (4.81), and was stable in the fourth and highest quartile (4.83). This difference between the quartiles was statistically significant (*p* = 0.005). A detailed characterization of the study population is presented in Table 1.

**Table 1:** Baseline characteristics of the study cohort

	n	Total study cohort	Patients with galectin-3 below the median split	Patients with galectin-3 above the median split	*P* value
Clinical data					
Age (years)	1260	66.7 ± 8.0	66.2	67.2	0.028
Married (%)	1207	66.9	66.1	67.7	0.563
Body-mass index (kg/m^2^)	1255	29.2 ± 4.9	29.3 ± 4.8	29.2 ± 5.0	0.661
Heart rate (beats/min)	1253	70.2 ± 12.0	70.6 ± 12.5	69.7 ± 11.6	0.157
Systolic blood pressure (mmHg)	1257	149.4 ± 21.3	149.1 ± 20.9	149.7 ± 21.8	0.650
Diastolic blood pressure (mmHg)	1257	84.1 ± 11.9	84.4 ± 11.8	83.9 ± 12.0	0.408
6-min walking distance (m)	1160	506.9 ± 111.5	509.1 ± 113.0	504.7 ± 110.1	0.502
Hypertension (%)	1260	88.7	89.0	88.3	0.672
Hyperlipidemia (%)	1260	45.1	44.8	45.3	0.871
Diabetes mellitus (%)	1260	28.3	29.3	27.4	0.470
Coronary heart disease (%)	1260	21.0	19.6	22.3	0.224
Previous myocardial infarction (%)	1260	10.3	8.6	12.0	0.044
Sleep apnea (%)	1260	6.5	7.5	5.5	0.166
Current smokers (%)	1259	11.2	11.6	10.8	0.884
LVEF (%)	1249	60.0 ± 8.1	59.9 ± 8.1	60.2 ± 8.1	0.537
ASE grade	1173	1.07 ± 0.65	1.1 ± 0.66	1.03 ± 0.63	0.001
Neurohumoral parameters					
Galectin-3 (ng/mL)	1260	11.7^a^ (9.9;14.1)^b^	9.9^a^ (8.7;10.7)^b^	14.1^a^ (12.8;16.3)^b^	< 0.001
CT-proAVP (pmol/L)	1220	4.1^a^ (2.6;7.6)^b^	4.1^a^ (2.6;7.8)^b^	4.2^a^ (2.6;7.5)^b^	0.757
NT-proBNP (pg/mL)	1160	101.4^a^ (52.5;193.1)^b^	78.6^a^ (43.3;151.9)^b^	125.6^a^ (65.1;256.1)^b^	< 0.001
MR-proANP (pmol/L)	1219	91.9^a^ (64.4;135.1)^b^	87.5^a^ (61.4;128.8)^b^	96.3^a^ (67.0;143.4)^b^	0.228
Psychometric variables					
HADS Anxiety	1230	5.1 ± 3.7	5.4 ± 3.9	4.8 ± 3.5	0.005
SF-36 Physical functioning	1237	72.0 ± 25.0	72.9 ± 25.1	71.0 ± 24.9	0.173
Medication use					
ACE inhibitor (%)	1239	45.0	44.2	45.8	0.570
AT_1_ receptor antagonist (%)	1239	19.0	20.3	17.8	0.256
Beta-blocker (%)	1239	50.0	54.0	46.0	0.005
Diuretics (%)	1239	49.7	51.4	48.1	0.245
Statins (%)	1239	31.7	31.7	31.7	0.993
ASS (%)	1239	35.6	36.7	34.5	0.399
Insulin and/or oral antidiabetics (%)	1239	21.1	20.7	21.6	0.671

Results are presented as means and standard deviations, percentages or frequencies, ^a^median, ^b^25^th^ and 75^th^ percentile. *ASE* American Society of Echocardiography, *CT-proAVP* C-terminal pro-arginine vasopressin, *HADS* anxiety subscale of the Hospital Anxiety and Depression Scale, *LVEF* left ventricular ejection fraction, *MR-proANP* mid-regional pro-atrial natriuretic peptide, *NT-proBNP* N-terminal pro-brain natriuretic peptide, *SD* standard deviation

**Fig. 1:**
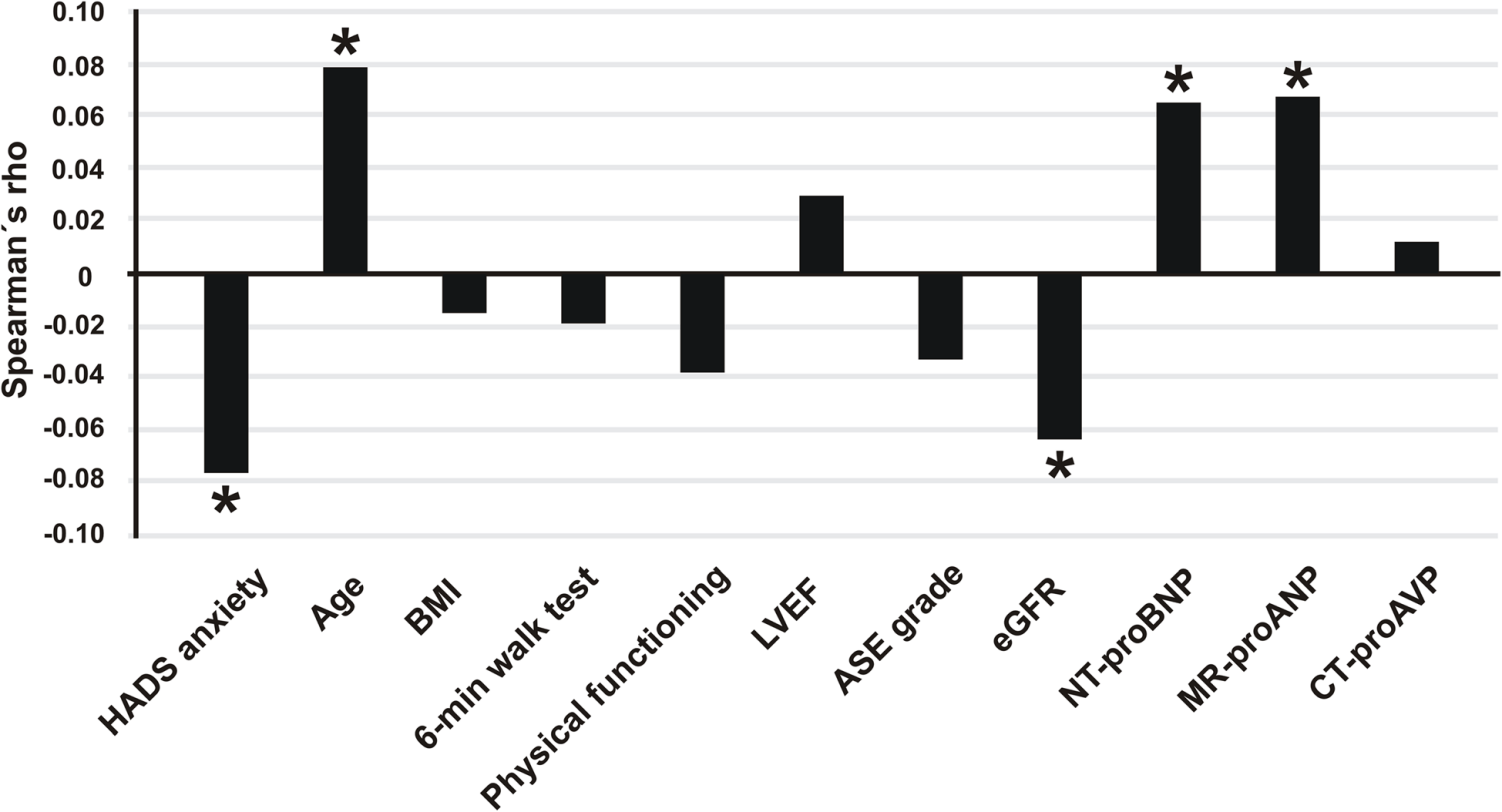
Spearman’s correlation coefficients of galectin-3 with self-rated HADS (Hospital Anxiety and Depression Scale) anxiety subscale, age, body-mass index (BMI), 6-min walking distance, SF-36 physical functioning, left ventricular ejection fraction (LVEF), American Society of Echocardiography (ASE) grade, estimated glomerular filtration rate (eGFR), NT-proBNP, MR-proANP and CT-proAVP. Abbreviations: NT-proBNP=N-terminal pro-brain natriuretic peptide, MR-proANP = mid-regional pro-atrial natriuretic peptide, CT-proAVP=C-terminal pro-arginine vasopressin

In Spearman’s correlation analysis, we found weak but significant positive correlations of galectin-3 with NT-proBNP (rho = 0.065, *p* = 0.003), MR-proANP (rho = 0.068, *p* = 0.012) and age (rho = 0.078, *p* = 0.005). There was also a significant negative correlation of galectin-3 with HADS anxiety (rho = -0.076, *p* = 0.008) and eGFR (rho = -0.063, *p* = 0.032), respectively (Fig. 1). There was no statistically significant correlation between galectin-3 and high sensitive C-reactive-protein (rho = -0.019, *p* = 0.513).

In a linear regression model adjusted for gender, age, BMI, eGFR, LVEF, ASE grade, 6-min walking test, and SF-36 physical functioning, we found that log-transformed galectin-3 concentrations independently predicted HADS anxiety (B = -2.493, 95%CI = -4.354–-0.632, *p* = 0.009) (Table 2, model 1). The coefficient of determination (R^2^) for this model was 0.115. A similar result was obtained for serum galectin-3, when the model was adjusted exclusively for age and sex (*p* = 0.009).

**Table 2:** Results from multivariate regression models in the Diast-CHF study group with HADS anxiety as the dependent variable

	B	95% confidence interval	β	*P* value
Model 1: Total model *p* < 0.001, adjusted R^2^ = 0.115, F = 15.051				
Galectin-3	-2.493	-4.354;-0.632	-0.080	0.009
Gender	0.605	0.128;1.082	0.081	0.013
Age	-0.054	-0.091;-0.018	-0.114	0.004
BMI	0.055	-0.114;0.004	-0.071	0.066
eGFR	0.008	-0.004;0.020	0.056	0.214
LVEF	0.021	-0.009;0.050	0.043	0.173
ASE grade	0.136	-0.219;0.490	0.024	0.453
6-min walking distance	0.002	-0.001;0.004	0.059	0.127
SF-36 physical functioning	-0.057	-0.068;-0.045	-0.355	< 0.001
Model 2: Total model *p* < 0.001, adjusted R^2^ = 0.108, F = 9.483				
Galectin-3	-2.413	-4.422;-0.404	-0.077	0.019
Gender	0.249	-0.323;0.821	0.033	0.394
Age	-0.053	-0.094;-0.013	-0.111	0.010
BMI	-0.034	-0.101;0.033	-0.043	0.318
eGFR	-0.002	-0.016;0.012	-0.013	0.809
LVEF	0.007	-0.025;0.039	0.015	0.669
ASE grade	0.200	-0.189;0.589	0.035	0.314
6-min walking distance	0.002	-0.001;0.004	0.044	0.287
SF-36 physical functioning	-0.056	-0.069;-0.043	-0.345	< 0.001
CT-proAVP	-0.913	-1.776;0.050	-0.078	0.038
NT-proBNP	-0.286	-1.212;0.640	-0.034	0.545
MR-proANP	-0.843	-2.665;0.979	0.052	0.364

Results from multivariate linear regression models with HADS anxiety scores as dependent variable and plasma galectin-3 as independent variable adjusted for gender, age, *BMI* body-mass index, *eGFR* estimated glomerular filtration rate, *LVEF* left ventricular ejection fraction, *ASE grade* American Society of Echocardiography, 6-min walking distance, SF-36 physical functioning and, optionally, CT-proAVP, and natriuretic peptides NT-proBNP and MR-proANP

Finally, we tested whether the inverse and significant association between galectin-3 and HADS anxiety remained stable, when additionally NT-proBNP, MR-proANP and CT-proAVP were included as confounders in the model. Data showed that, also in this model, galectin-3 was a significant and independent predictor for anxiety (B = -2.413, 95%CI = -4.422–-0.404, *p* = 0.019) (Table 2, model 2). However, the natriuretic peptides MR-proANP and NT-proBNP concentration failed to predict self-assessed anxiety. In contrast, the CT-proAVP concentration was inversely and significantly associated with anxiety (B = -0.913, 95%CI = -1.776–-0.050, *p* = 0.038) (Table 2, model 2).

## Discussion

The main finding of this post-hoc analysis from the Diast-CHF study is that, in a cohort of patients with cardiovascular risk factors, the plasma concentration of galectin-3 is inversely correlated with self-assessed anxiety. This relationship between galectin-3 and HADS anxiety was weak but remained stable when, in a multivariate model, clinical variables indicative of physical impairment were added as putative confounders. Our data demonstrate that, independently of natriuretic peptides and the biomarker CT-proAVP, higher levels of circulating galectin-3 predict less anxiety, suggesting that the β-galactoside-binding protein galectin-3 may modulate neuronal pathways engaged in the regulation of anxiety. Our observation extends previous findings from animal and clinical studies suggesting that galectin-3-mediated pathways are implicated in a variety of processes associated with myocardial fibrogenesis, tissue repair, inflammation, myofibroblast proliferation, and ventricular remodeling. However, our cross-sectional data do not prove a causal effect of galectin-3 on anxiety. Higher anxiety might also lead to suppressed production and/or release of galectin-3 or both could depend on an unknown third factor not identified in our analyses.

Psychological stressors, especially those that produce a state of fear, commonly lead to relevant anxiety, which concurrently explains why stress and anxiety share overlapping physiological reactions. The anti-inflammatory effects of cortisol usually associated with increased stress levels and anxiety may have an overall suppressive effect on the synthesis and subsequent release of galectin-3 in the circulation [45]. In line, we observed in our study population that decreased circulating galectin-3 concentrations are linked to higher levels of anxiety, which may in turn have resulted from a chronically elevated psychological stress level. Thus, our main finding of an inverse relationship between self-rated anxiety and galectin-3 secretion into the circulation may not be completely unexpected.

In an important study in mice, it was showed that a single episode of immobilization stress reduces galectin-3 expression in the liver, spleen and in macrophages. These animal experiments suggest that galectin-3 is involved in the overall regulation of physiological responses to psychological stress [25]. Untreated mice lacking galectin-3 expression showed anxiogenic behaviors compared to wild-type animals, and this was associated with lower brain-derived neurotrophic factor (BDNF) gene expression and immunoreactivity in hippocampal tissue (predominantly in the CA1 region) [33]. Interestingly, hippocampal gene expression of the GABA-A receptor subunits 2 and 5 was attenuated in the transgenic mice.

It has been shown that galectin-3 overexpression in hypoxic and nutrient-deprived microenvironments promotes a cell survival adaptive program under stressing conditions [46]. In contrast, knockdown of galectin-3 expression led to reduced NF-κB promoter activity and secretion of interleukin-8 and attenuated inflammatory response in the peritoneal cavity, further confirming its activation in inflammatory tissue microenvironments [47]. Complexes with galectin-3 have been described as ligands activating Toll-like receptors and mediating NF-κB signal transduction [25]. Oligomers of galectin-3 interact with a variety of cell surface glycoproteins and serve as scaffolds for the spatial organization of signaling receptors. Galectin-3 is a mediator of microglia responses in injured brain regions promoting cell proliferation and cell survival [48].

However, it is unknown whether or not galectin-3 expressed in the brain has any functions in mediating anxious behavior in humans. Beside its prognostic value for heart failure patients [20, 49, 50], our data from the Diast-CHF study suggest an unknown mechanism of galectin-3 in the regulation of anxiety also in humans. Thus, this galactoside-binding lectin may be added to the growing list of peptides functioning as prognostic biomarkers of heart failure (NT-proBNP, NT-proANP, MR-proANP, CT-ET-1, CT-proAVP, and MR-pro-adrenomedullin) that, in addition, negatively associate with anxiety [13–16, 51, 52].

The present post-hoc analysis has important methodological limitations, which result mainly from the observational and cross-sectional design of the study. No causal inferences can be made from our findings, as longitudinal data were not available. Moreover, we noted that the described effects are generally small and there are no direct implications for routine clinical care from our data. It remains to be tested whether the main result from this study can be confirmed in other populations with or without cardiac risk factors. In addition, it is unclear whether our findings for galectin-3, CT-proAVP and natriuretic peptides, which were obtained from a single cohort, may be generalized to other patients’ samples with more severe heart failure symptoms. Future research will have to show whether indeed galectin-3 may attenuate anxiety-related responses and thus protects the heart from the adverse cardiac effects of psychosocial stress.

## Conclusion

In patients with cardiovascular risk factors, serum galectin-3 is linked to anxiety and this relationship is independent of physical impairment and serum concentrations of natriuretic peptides. The association between galectin-3 and HADS anxiety is weak and remains stable in multivariate models adjusted to clinically relevant confounders. Galectin-3 is added to the growing list of prognostic biomarkers of heart failure negatively associated with anxiety.

## Data Availability

Data from participants who agreed to the public distribution of data are available from the corresponding author upon reasonable request.
